# Polymorphism Identification in the Coding Sequences (ORFs) of the Porcine Pregnancy-Associated Glycoprotein 2-like Gene Subfamily in Pigs

**DOI:** 10.3390/genes15091149

**Published:** 2024-08-31

**Authors:** Martyna Bieniek-Kobuszewska, Grzegorz Panasiewicz

**Affiliations:** 1 Voivodeship Sanitary-Epidemiological Station in Olsztyn, Laboratory of Epidemiological and Clinical Research, Department of Virology and Serology, Zolnierska Str. 16, 10-561 Olsztyn, Poland; martyna.bieniek3@gmail.com; 2 Department of Animal Anatomy and Physiology, Faculty of Biology and Biotechnology, University of Warmia and Mazury in Olsztyn, Oczapowskiego Str. 1A, 10-719 Olsztyn, Poland

**Keywords:** amino acid substitution, InDel, pig, pPAG, SNP

## Abstract

Pregnancy-associated glycoproteins (PAGs) are a polygenic family with many scattered genes and pseudogenes resulting from the duplication or fusion of a pseudogene with expression beginning in the trophoblast during the peri-implantation period and continuing in the trophectoderm. In this study, single-nucleotide polymorphism (SNP) and insertion/deletion (InDels) in the open reading frame (nine exons) of crossbreed pigs are reported for the first time. Novel SNPs/InDels were researched using genomic DNA templates isolated from the leukocytes of crossbreed pigs (N = 25), which were amplified, gel-out-purified, and sequenced. Sixteen SNPs and one InDel (g.6961_6966 Ins TGCCAA) were identified in the crossbreed pigs. In silico analysis revealed that among 16 SNPs, only 10 SNPs cause amino acid (aa) substitutions, and InDel codes asparagine (N^298^) and alanine (A^299^). The results provide a novel broad-based database (main pattern) that will be critical for future research into the possible correlations between the SNP genotypes of the *pPAG2-L* subfamily in pigs of various breeds whose reproductive traits are known.

## 1. Introduction

The pregnancy-associated glycoprotein gene family (*PAGs*) constitutes a multigene family characterized by a large number of dispersed genes and pseudogenes. The diversity of *PAGs* results from the duplication or fusion of the progene. Meanwhile, the variable reproductive abilities of animals may result from the positive selection of these genes during species evolution [1,2,3]. It has been shown that, regardless of the type of placenta, the expression of the PAG family is limited only to trophoblastic cells and the trophoderm (chorionic epithelium). As mentioned, the expression of the mRNA of *PAGs* in the placenta begins in the peri-implantation period and continues in the further stages of the development of this organ. The expression of the mRNA of *PAGs*, temporarily specific for a given stage of placenta development, has been identified in cattle, sheep, pigs, horses, goats, European bison, white-tailed deer, some camelids (alpaca, dromedary, and Bactrian camel), Eurasian beaver, and humans [4,5,6,7,8,9,10,11,12,13,14,15,16,17,18,19,20,21,22].

The first complementary DNA (cDNA) of the *PAG* family genes was described in 1991. These were the genes *bPAG1* and *oPAG1* in cattle and sheep, respectively. In the following years, the cDNAs of *pPAG1* and *pPAG2*, i.e., the first known *PAG* genes in pigs, were cloned. Among the genes identified and deposited in the international GenBank database, the cDNA of *PAGs* (at least 75 differentiated cDNAs) have been cloned, mainly in domestic mammals: cattle, sheep, pigs, horses, goats, cats, mice, and humans [4,7,9,10,12,16,21]. However, for wild species (e.g., zebra, white-tailed deer, water buffalo, bison, and wapiti), only 35 cDNAs of *PAG* genes have been cloned [23,24,25]. The significantly smaller biological material numbers and the difficulties that are encountered in the proper placement of the placenta determine the isolation of high-quality mRNAs. Only high-quality mRNAs can enable the effective cloning of full-length cDNA, including both coding sequences (ORFs) and noncoding regions (5′UTR and 3′UTR) [7,12,16]. Any newly cloned cDNA of the *PAGs* can be used to produce recombinant proteins. It is necessary to undertake research that aims to build an understanding of the functions of PAG proteins. Previous studies have shown that they are crucial in the period around implantation and in subsequent stages of pregnancy in placentals, including wild species.

The PAG family code proteins are classified as glycoproteins. The pregnancy-associated glycoproteins known so far have been classified into the family of aspartyl proteinases (APs), EC 3.4.23 [26]. Such proteins are widely distributed among vertebrates [26,27], as well as plants, fungi, and retroviruses [28,29]. The prevalence of APs and their proteolytic activity, preserved in evolution, indicates the extremely important role of the protein family in the proper functioning of even very phylogenetically distant organisms. In vertebrates, the AP family includes proteolytic enzymes: pepsin A, C, and F, aspergillopepsin A, candidopepsin, renin, chymosins, and lysosomal enzymes such as cathepsins [27,30]. The AP family also includes human napsines and parasitic enzymes: histo-aspartic proteinase (HAP) and plasmepsins [31,32]. The conservativeness of the AP family is related to the evolutionally preserved structure of the active center, which, regardless of the type of enzyme, contains two aspartic acid residues. Since the catalytic center constructed in this way enables the hydrolysis of peptide bonds, the products of the PAG gene may also have proteolytic activity [16].

The complete exon–intron organizational structure has only been defined for six *PAG* genes: two in cattle [18,33] and one in pigs [34], horses [8], Eurasian beavers [21], and humans [22]. The structure of these genes is conservative and includes nine exons and eight introns (named A-H) of relatively similar lengths. In their structure, the *PAG* genes have exon–intron connections (5′ and 3′) with the sequence gt-ag, which are characteristic not only for AP but also for most other mammalian genes [16,34].

Mammalian reproductive abilities may be associated with the positive selection of *PAG* genes during evolution. Therefore, the study of this family, including the determination of the number of *PAG* genes in particular placental species and the determination of whether this abundance is species-specific, seems to be crucial in understanding reproductive regulation. The research results indicate that, in the genome of the placental mammal species analyzed so far, the *PAG* family consists of many genes. For example, there may be over 100 *PAG* genes in domestic ruminants [5]. In turn, at least eight *pPAG2-like* genes (*pPAG2-L*) have been demonstrated in pigs, while the number of *pPAG1-like* genes (*pPAG1-L*) is not yet known [34]. *PAG-like* genes (*PAG-L*) have also been found in the genomes of wild Artiodactyla species, including elk, buffalo, yak, white-tailed deer, giraffe, hippopotamus, impala, gnu, many other antelopes, and a Ursidae representative, i.e., the great panda. Evolutionary and physiological factors may justify/explain the emergence of such a rich and, at the same time, possibly differentiated genomic number of genes of the PAG family [5,16].

All polypeptide precursors of PAGs, encoded by the cloned cDNAs known to date, are included in two subfamilies, known as catalytically active or catalytically inactive. Belonging to a given subfamily depends on the preservation of the conservative amino acid sequence or the occurrence of substitutions within two domains, with the first and the second domains making up part of the active center forming the catalytic pocket of a given glycoprotein [35].

In the domestic pig, full-length or partial/shortened ORF sequences for catalytically active *pPAG2-L* (Acc. No. L34361, AF272734, AF272735, AY373029, and AY775784) and potentially inactive *pPAG1-L* (Acc. No. L34360, AF315377, and AY188554) members have been identified and classified as the AP family [7,12]. Both subfamilies are characterized by 71–74% sequence homology and about 64% polypeptide precursor homology (acc. Blast/NCBI). In silico analysis of the cloned PAG ORFs revealed 376–388 aa coding, placentally expressed polypeptide precursors, or shorter forms with several missing exons (258–346 aa); these are activated by the removal of the conserved 15 aa signal peptide and 37–38 aa blocking pro-peptides (as the AP family), or they are modified by post-transcriptional glycosylation, which leads to there being varied catalytically secretory proteins [4,7,8,10,12].

All isolated isoforms of native and recombinant pregnancy-associated glycoproteins (PAGs) have been applied as standards required for prenatal diagnosis of early pregnancy and monitoring fetal mortality, with the use of radioimmunology assay (RIA) or enzyme-linked immunosorbent assay (ELISA) methods in domestic and wild ruminants (see [16]). The cloned cDNAs of the *PAGs* constitute the basis of various patents to prepare non-commercial [36] and commercial BioPRYN^®^ diagnostic tests [37]. To date, studies have been conducted on variable PAG concentrations in the peripheral blood or in the milk of domestic ruminants using RIA and ELISA [38,39,40,41,42]. Similar studies have also been carried out on wild animals such as reindeer [43,44] and water buffalo [45,46]. The concentration of PAGs in the peripheral blood of pregnant ruminants was also used to identify the gender of fetuses or to determine a single or plural pregnancy [16], as well as to detect abnormalities during pregnancy in cattle [47,48,49] and to predict miscarriage after embryo transfer [50]. However, the polymorphic *pPAG2-L*, with identified SNPs and InDels [51,52], was applied in a pioneering comparative genomics study concerning the correlation of SNPs with reproductive traits in hybrid sows [53]. Large-scale gDNA and cDNA sequencing is a source of an ever-increasing set of available markers. However, the molecular substrate characteristics of reproduction features are still poorly understood. The number of identified SNPs/InDels in the pig genome (version Sscrofa11.1), referred to as quantitative trait nucleotides (QTN), enables a phenotype effect analysis for economically important traits, i.e., quantitative trait loci (QTL) [54,55]. The current release of the Pig QTLdb contains 48,875 QTLs/associations “http://www.animalgenome.org/cgi-bin/QTLdb/index (accessed on 5 July 2024)” [56]. This study aimed to identify missing, previously exonic SNPs within the entire ORF sequence (nine exons) of *pPAG2-L* with gDNA templates in crossbreed pigs. Further studies are needed to explore the potential association of a genotype/SNP with reproductive effectiveness for the development of a new genetic test.

## 2. Materials and Methods

### 2.1. Animals, Genomic DNA (gDNA) Templates, and PCR Amplification

This study was conducted according to the Polish Act of 15 January 2015 on the protection of animals used for educational or scientific purposes and directive 2010/63/EU of the European Parliament and of the Council of 22 September 2010 on the protection of animals used for scientific purposes (Journal of Laws Dz.U. 2015 No. item 266); therefore, the consent of the competent ethics committee for experiments on animals was not required.

The blood samples were harvested (*post mortem*) into 50 mL conical tubes containing K2EDTA from crossbreed pigs (Polish Large White × Polish Landrace × Duroc × Pietrain; from a private farm in the Warmian-Masurian Voivodeship in Poland; N = 25) slaughtered under commercial conditions. All blood samples were centrifuged (1500 rpm/20 min/4 °C) and used as the material for the isolation of gDNA templates (Sherlock AX, A&A Biotechnology, Gdansk, Poland). The *pPAG2-L* amplicons were generated by PCRs using primers (Table 1) designed based on pPAG2 [32] and *pPAG2-L* [8,27]. The PCR mix with a final volume of 20 μL contained 0.42 μL of dNTP, 0.4 μL 25 mM of MgCl2, 2 μL of 10× Buffer B, 0.4 μL of JumpStart^TM^Taq DNA Polymerase (Sigma-Aldrich, St. Louis, MO, USA), 0.7 μL of each primer (100 ng/μL), and 200 ng templates (gDNA of the crossbreeds). The PCR reactions were run for initial denaturation at 94 °C/3 min, 40 cycles of 94 °C/1 min, 62 °C/2 min, 72 °C/2 min, and final elongation at 72 °C/7 min, as previously described [51].

### 2.2. Sequencing and SNPs/InDels Identification in the ORF of the pPAG2-L

The obtained *pPAG2-L* amplicons were electrophoretically separated in 1% gels, cut out from agarose gel by Gel-X Excision Tips (BIOCOMdirect, 6 Lomond Crescent, Bridge of Weir, UK), purified (GenElute^TM^ Gel Extraction Kit, Sigma-Aldrich, St. Louis, MO, USA), and then sequenced in both sense/antisense directions (3130 Genetic Analyzer, Applied Biosystems, Waltham, MA, USA). The amplicon labeling was performed with the BigDye Terminator v3.1 Cycle Sequencing Kit (Applied Biosystems, Waltham, MA, USA) with modifications. Briefly, each labeling amplification mix (10 μL) contained 1 μL (approximately 5 ng) of appropriate amplicon template, 2 μL of Ready Reaction Mix, 3 μL of BigDye Terminator v1.1/3.1 Sequencing buffer (5×), and 4 μL of H_2_O. The modified labeling conditions were as follows: initial denaturation (at 96 °C for 1 min) and then 25 cycles of 96 °C/10 s, 50 °C/5 s, 60 °C/4 min. Labeled amplicons were purified with the BigDye X Terminator Purification Kit (Applied Biosystems, Waltham, MA, USA) and separated in capillaries filled with POP-7™ polymer. The sequences were verified on chromatograms with the use of Finch TV software (Geospiza, Inc., Denver, CO, USA) and analyzed in DNASIS^®^ MAX v3.0 (Hitachi, Santa Clara, CA, USA) and in the NCBI BLASTn database using Megablast or Blastn. All SNPs were encoded according to IUPAC (International Union of Pure and Applied Chemistry).

## 3. Results

**Figure 1:**
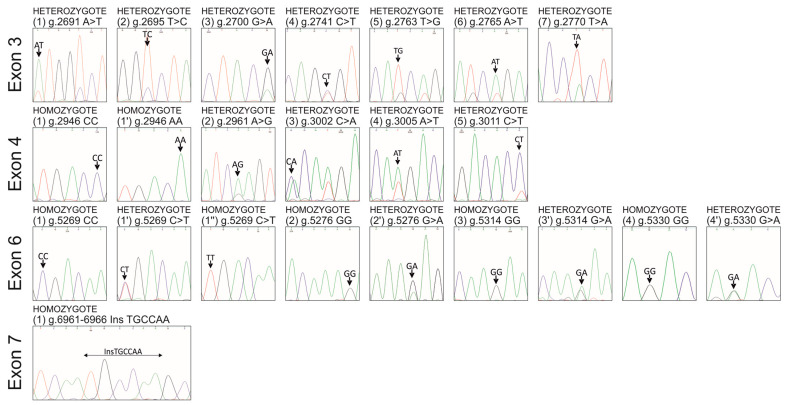
Representative chromatograms of identified SNPs and InDel (arrows) in homozygotes and heterozygotes within the *pPAG2-L* subfamily in crossbreed pigs.

**Figure 2:**
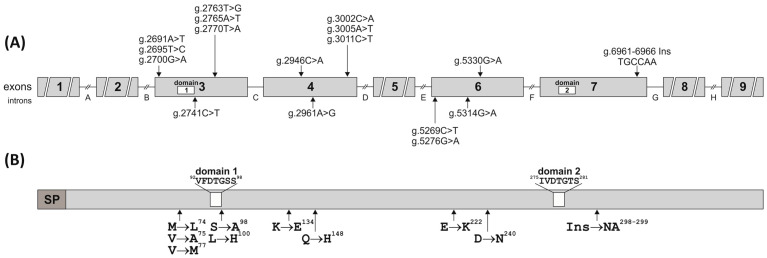
Schematic localization of the SNPs and InDel (**A**) and amino acid substitutions (**B**) in the pPAG2-L subfamily examined in the crossbreed pigs. White boxes indicate the location of domains 1 and 2 in the nucleotide and amino acid sequences of pPAG2-L (SP—signal peptide).

This is the first report describing SNPs/InDels identification in all nine exons of the *pPAG2-L* in crossbreed pigs. Due to the identified polymorphism relative to the consensus *pPAG2* (L34361) [32], novel SNPs (Figure 1 and Figure 2 and Table 2) identified in exons, including 3 (U39199), 4 (U39199), 6 (U41424), and 7 (U39762), were named *pPAG2-L*.

### 3.1. Seven Novel SNPs in Exon 3 of the pPAG2-L in the Crossbreed Pigs

Seven SNPs within the entire exon 3 (U39199) of *pPAG2-L* in crossbreed pigs were identified (Table 2, Figure 1 and Figure 2). In silico analysis (Table 3) showed that among seven heterozygous SNPs (px = 1.0), six SNPs represent missense mutations, which determine aa substitutions in the *pPAG2-L* polypeptide precursor: M→L^74^ (g.2691A>T); V→A^75^ (g.2695T>C); V→M^77^ (g.2700G>A); S→A^98^ (g.2763T>G; g.2765A>T); L→H^100^ (g.2770T>A). Only one SNP, i.e., g.2741C>T, does not cause substitution in the pPAG2-L precursor because both codons AGC and AGT encode serine (S^90^ *italics* in Table 3).

All the substitutions were identified in the neighborhood of the characteristic PLRN sequence (67–70 aa among 387 aa; the numbering of the precursor including 15 aa signal peptide; Figure 2), which is conservatively preserved in most existing precursors of the PAG family across various species during evolutionary processes. It should be noted that two SNPs (g.2763T>G and g.2765A>T) cause an aa substitution Ser→Ala^98^ (**T**C**A**→**G**C**T**) in domain 1 of the precursor (Table 3, Figure 2), which constitutes half of the active center formed by the fusion with domain 2.

### 3.2. Five Novel SNPs in Exon 4 of the pPAG2-L in the Crossbreed Pigs

Five SNPs were identified within the entirety of exon 4 (U39199) of the *pPAG2-L* gene subfamily in crossbreed pigs (Table 2). All identified SNPs have different frequencies of alleles in the range of p_x_ = 0.07–1. Among five SNPs (Table 2), four heterozygous SNPs (p_x_ = 0.79–1) were found, whereas one SNP (g.2946C>A) was identified in homozygous genotypes CC (p_x_ = 0.33) or AA (p_x_ = 0.67). In silico analysis (Table 3) revealed that among five SNPs, only two SNPs (g.2961A>G (K→E^134^); g.3005A>T) caused the missense mutation (Q→H^148^). Three other SNPs, i.e., g.2946C>A, g.3002C>A, and g.3011C>T, constitute synonymic mutations (*italics* in Table 3) because both codons **C**GG and **A**GG encode Arg^129^, GG**C** and GG**A** encode Gly^147^, and AC**C** and AC**T** encode Thr^150^.

### 3.3. Four Novel SNPs in Exon 6 of the pPAG2-L in the Crossbreed Pigs

Four SNPs were identified within the entirety of exon 6 (U41424) of the *pPAG2-L* gene subfamily in crossbreed pigs (Table 2). All identified SNPs have different frequencies of alleles in the range of p_x_ = 0.1–0.9. Among four SNPs (Table 2), the domination of three homozygous SNPs (p_X_ = 0.56–0.88) was found, whereas one SNP (g.5269C>T) was characterized by three genotypes: CC (p_x_ = 0.08), CT (p_x_ = 0.63), and TT (p_x_ = 0.29). In silico analysis (Table 3) revealed that among four SNPs, only two SNPs caused the missense mutation: E→K^222^ (g.5276G>A) and D→N^240^ (g.5330G>A). Two other SNPs constitute synonymic mutations: g.5269C>T (Ser^219^) and g.5314G>A (Lys^235^; *italics* in Table 3).

### 3.4. Novel InDel in Exon 7 of the pPAG2-L in the Crossbreed Pigs

This study identified six nt InDel (g.6961_6962InsTGCCAA) throughout the entire exon 7 (U39762) of the *pPAG2-L* gene subfamily in crossbreed pigs (Table 2). In silico analysis (Table 3) revealed that the 6 nt InDel coding asparagine (N^298^) and alanine (A^299^) is the same as ORF present in pPAG4, pPAG6, pPAG8, and pPAG10 but absent in *pPAG2*. The insertion NA^298−9^ has been identified in the neighborhood of the COOH domain (^260^IVDTGTS^266^, numbering after precursor degradation lacking the 15 aa signal peptide), which forms half of the pocket comprising the active center of AP (Figure 2).

## 4. Discussion

This is a pioneering study identifying 16 SNPs and 1 InDel in the ORF of the *pPAG2-L* in the crossbreeds required for examination of genetic variation in various breeds and provides a key pattern for the identification of polymorphism in reproductive animals with known reproductive traits. No SNP loci were identified in exons 1, 2, 5, 8, and 9 of the *pPAG2-L* in a crossbreed, implying the occurrence of conservative regions of homozygosity (ROHs). Generally, the locations of SNPs are not randomly distributed throughout the genome but are concentrated in clusters and associated with hypermutation regions, known as “hot spots” [57]. However, in humans, the dispersion of SNPs along the chromosomes often affects crossing-over, which mainly occurs in ROHs [58,59]. The reduction in the polymorphic regions could depend on the bottleneck effect and the rate of recombination in human populations [60]. It has been found that the African population (A) has a much higher level of allelic heterozygosity than the European (E), and the proportions of missense mutations are substantially higher in E—55.4%, than in A—47.0% [61]. The comparison of the Asian and European populations of *Suidae* showed the highest hypermutation in heterozygous regions [62].

The data obtained in the current study showed that the SNPs identified in exon 3 of *pPAG2-L* ORF cause a V → M^77^ substitution (Table 3), which occurs in the conserved 1-NH2 domain (^77^VFDTGSS^83^) forming half of the binding site. Substitutions in the 1-NH2 domain (77–83 aa) suggest a loss of catalytic activity of PAG proteins, although it is worth noting that numerous mutations in this domain of the HAP (histo-aspartic proteinase) enzyme of *Plasmodium falciparum* (subtropical malaria parasite) do not result in a loss of enzymatic activity or inhibition of the development cycle [31,32]. All aforementioned aa substitutions were identified in the neighborhood of the characteristic PLRN sequence (67–70 aa, the numbering of the precursor including the 15-aa signal peptide), conservatively preserved in the majority of existing PAG family precursors in various animal species [16]. However, no SNPs were identified at the potential N-glycosylation site of asparagine (N^119^). In contrast, a conservatively preserved PLRN sequence was found in the pPAG2-L precursor, similar to numerous bPAG genes that contain the characteristic N-acetylgalactosamine transferase domain (PLR). In equines, the ePAG precursor is changed into PMRN, but in the potentially catalytically inactive precursors of the pPAG1-L subfamily (pPAG1, −3 and −5), it is modified to RLWN [12].

All of the four SNPs in exon 6 of the *pPAG2-L* identified in crossbreed pigs (Table 2 and Table 3) have also been identified in the JSR Hirschmann hybrids (Hrn) with known reproductive traits [53], which confirms the usefulness of the genetic pattern for the examination of polymorphism in various breeds. Interestingly, among the eight SNPs identified within exon 6 in Hrn, one specific SNP diplotype (g.5269C>T/5361G>C) revealed an important correlation between the *PAG* SNPs with reproductive traits. Significant associations (*p* ≤ 0.05) were identified in the number of piglets born alive (12.71 ± 0.47 per litter) with the 5269CC/5361GC genotype compared to 5269CT/5361GG (11.39 ± 0.22 per litter). These results introduce a novel database necessary for further research on potential correlations between SNP genotypes of the pPAG2-L subfamily in sows of various breeds. This will enable the pre-selection of young future progenitors (females and males) and the breeding of the best reproductive animals. It also qualifies *pPAG2-Ls* as candidate genes for the main QTLs related to meat and carcass quality, health, and reproductive traits.

The authors’ previous data [51,52] show that the 3′ region of *pPAG2-L*, coding the 2-COOH domain, which constitutes half of the catalytic binding center, is less polymorphic. The current research confirms previous findings. In exon 7, 6 nt InDel (g.6961_6962InsTGCCAA) encoding asparagine (N^298^) and alanine (A^299^) was identified. This sequence is identical to the ORF sequence of *pPAG4*, *pPAG6*, *pPAG8,* and *pPAG10* but not to *pPAG2*. No *SNP* loci were identified in exons 8 and 9 of *pPAG2-L* in crossbreed pigs, which suggests the occurrence of ROHs. However, the 3′ region of the *pPAG* represents heterologous sequences that distinguish the *pPAG1-L* and *pPAG2-L* subfamilies, and therefore, this region was used for the various microarray productions. The analysis of the Affymetrix GeneChip microarray, consisting of the mRNAs of genes expressed in the placenta (75 and 90 dpc) in the Large White and Chinese pig (*Erhulian*), revealed 7.14-times lower expressions of *pPAG3* and 7.6-times lower expressions of the *pPAG6* [63]. The reduced expression of the *pPAG1-L* and *pPAG2-L* subfamilies confirms previous findings from a study that used the Northern method to analyze transcripts [7]. In the study, it was necessary to amplify much more purified poli(A)+RNA transcripts (60, 75, and 90 days post coitum; dpc) instead of total RNA (10–30 dpc). The subsequent comparison of results from the mRNA microarray (Agilent Technologies, Santa Clara (CA), USA) concerning transcripts from the endometrium of pregnant (15–16 dpc) and cyclic (15–16 days) pigs reveals increased expression of 266 genes (including *pPAG3* 1.25x) and reduced expression of 333 genes (including *pPAG6*) during pre-implantation [64]. It should be clarified that this matrix consists of a *pPAG3* probe that hybridizes to the 858–917 bp region (whole exon 7 and 2 nt of exon 8), while the pPAG6 probe hybridizes to the 3′UTR region (1301–1360 bp) preventing the proper study of transcript expression. It should be noted that since the *PAG* family is expressed in the trophoblast, it could be used as a marker to detect trophoblast residues, which penetrate endometrial tissue during the formation of the folds that increase the placenta surface [7]. The use of microarrays in the cattle *revealed an increase in the expression* of 16 mRNAs from the bovine PAG gene family (*bPAGs*), mainly *bPAG6* and *bPAG19* (21 dpc). This indicates the participation of the *bPAGs*, lactogen, and prolactin-related proteins (PRPs) in the apposition, fusion, and adhesion of the embryonic trophoblast to the endometrial epithelium [65].

DNA polymorphism is widely recognized as playing a key role in the development of new genetic markers, while allele differentiation in the regulatory regions has a strong effect on gene expression. Therefore, further research using polymorphic *pPAGs* in female and male pigs with documented reproductive traits should provide valuable information for mapping quantitative trait loci (QTLs).

## 5. Conclusions

This study enhances the general understanding of the porcine genome. The identification of SNPs in the pPAG2-L subfamily in crossbreed pigs (porkers) could serve as a valuable foundation for economically viable piglet pre-selection. Analyzing the *pPAG2-L* subfamily in crossbreed pigs enables the identification of dominant SNPs, which are present even in individual animals. Consequently, therefore, these data are significant for future research. Further investigation with a larger breeding sample is needed to establish additional correlations between SNP genotypes and reproductive traits.

## Figures and Tables

**Table 1:** Sequences of the primers used during PCR amplification of the various fragments of the *pPAG2-L* in the crossbreed pigs.

Exons Studied	Primer	Sequence
Ex1	F: pr500se	5′GACTGCCTGTGTCTTACT3′
	R: IntrAas	5′GACTGTCAGGAATGATGGCA3′
Ex1	F: sensATG	5′TGGASCCAGGAAAGAAGCATG3′
	R: IntrAasSr	5′GGATGAGAGATGGAGCCAGGAG3′
Ex2	Ex2se	5′TACAACATGATCCAGAATCT3′
	R: Ex3asSR	5′GATGAGSCGGTGTCAAAKAYGAC3′
Ex3	F: IntrBsedo3	5′GCTGCTCCAATGAGGCACTGGT3′
	R: Ex4as	5′GTCATGGAAGGTGCTGGAGTG3′
Ex3,4	F: Ex3seP	5′TGGTCTACGTGGGCAACATCA3′
	R: IntrDasSr	5′CCTAGAGTAAGAATGCACTTAGCAG3′
Ex4	F: Ex4pr	5′CCATCTACTGCAAAAGCAAG3′
	R: Ex5as	5′GGGATAGGCCAGGCCCAGGA3′
Ex5	F: IntrDsedo5	5′AGG AGCGGCCGTGGGTTGAAAT3′
	R: IntrEas	5′CTTCCTTATCGAATCCGTCAGCG3′
Ex6	F: IntrEsedo6	5′GACTCTCTCAGTAGGCTGATTGC3′
	R: Ex8as	5′GACRTTGTTAATGGTGAAGA3′
Ex6	F: IntrEsedo6	5′GACTCTCTCAGTAGGCTGATTGC3′
	R: IntrFasGap	5′TAGCCTGGAGCAAAGTGGTAATTCATTC3′
Ex7,8,9	F: IntrFsedo7	5′GAATGAATTACCACTTTGCTCCAGGCTA3′
	R: Ex8as	5′GACRTTGTTAATGGTGAAGA3′
	F: nEx7se	5′GCTGCCAGGCCATCKTGGATA3′
	R: pagC1155	5′CAGGCCAATCCTGTTCTGTCCT3′

**Table 2:** Identification of SNPs, genotypes, and allele frequency (px) within exons of the *pPAG2-L* subfamily in crossbreed pigs.

SNP Locus (IUPAC Code) *	SNP Locus acc. DNASIS **	Genotype Frequencies (p_x_)					
		Genotype	p_x_	Genotype	p_x_	Genotype	p_x_
**Exon 3**							
g.2691A>T (W)	g.1A>T	AA	0	AT	1.0	TT	0
g.2695T>C (Y)	g.5T>C	TT	0	TC	1.0	CC	0
g.2700G>A (R)	g.10G>A	GG	0	GA	1.0	AA	0
g.2741C>T (Y)	g.51C>T	CC	0	CT	1.0	TT	0
g.2763T>G (K)	g.73T>G	TT	0	TG	1.0	GG	0
g.2765A>T (W)	g.75A>T	AA	0	AT	1.0	TT	0
g.2770T>A (W)	g.80T>A	TT	0	TA	1.0	AA	0
**Exon 4**							
g.2946C>A (M)	g.48C>A	CC	0.33	AC	0	AA	0.67
g.2961A>G (R)	g.63A>G	AA	0.21	AG	0.79	GG	0
g.3002C>A (M)	g.104C>A	CC	0	AC	1.0	AA	0
g.3005A>T (W)	g.107A>T	AA	0.07	AT	0.93	TT	0
g.3011C>T (Y)	g.113C>T	CC	0.07	CT	0.93	TT	0
**Exon 6**							
g.5269C>T (Y)	g.1C>T	CC	0.08	CT	0.63	TT	0.29
g.5276G>A (R)	g.8G>A	GG	0.64	AG	0.36	AA	0
g.5314G>A (R)	g.46G>A	GG	0.85	AG	0.15	AA	0
g.5330G>A (R)	g.62G>A	GG	0.9	AG	0.1	AA	0
**Exon 7**							
g.6961–6966 Ins TGCCAA	g.121–126 Ins TGCCAA	TGCCAA	1.0	-	-	-	-

(*) Numbering of SNPs submitted in dbSNP/NCBI database; (**) Numbering according to the sequence of exons 3, 4, 6, and 7 (U39199, U39199, U41424, and U39762 GenBank, respectively).

**Table 3:** In silico analysis of SNPs within exons coding amino acid (aa) substitutions of the polypeptide pPAG2-L precursors.

SNP Locus (IUPAC Code) *	*pPAG2* Codon	Consensus/Code [aa]	Codon with SNP	Localization of Substitution [aa] **	Substitution/Code [aa]
**Exon 3**					
g.2691A>T (W)	**A**TG	Met (M)	**T**TG	74	Leu (L)
g.2695T>C (Y)	G**T**C	Val (V)	G**C**C	75	Ala (A)
g.2700G>A (R)	**G**TG	Val (V)	**A**TG	77	Met (M)
g.2741C>T (Y)	*AG**C***	*Ser (S)*	*AG**T***	*90*	*Ser (S)*
g.2763T>G (K)	**T**CA	Ser (S)	**G**CT	98	Ala (A)
g.2765A>T (W)	TC**A**	Ser (S)	GC**T**	98	Ala (A)
g.2770T>A (W)	C**T**C	Leu (L)	C**A**C	100	His (H)
**Exon 4**					
g.2946C>A (M)	***C*** *GG*	*Arg (R)*	***A*** *GG*	*129*	*Arg (R)*
g.2961A>G (R)	**A**AG	Lys (K)	**G**AG	134	Glu (E)
g.3002C>A (M)	*GG**C***	*Gly (G)*	*GG**A***	*147*	*Gly (G)*
g.3005A>T (W)	CA**A**	Gln (Q)	CA**T**	148	His (H)
g.3011C>T (Y)	*AC**C***	*Thr (T)*	*AC**T***	*150*	*Thr (T)*
**Exon 6**					
g.5269C>T (Y)	***C*** *GA*	*Ser (S)*	***T*** *GA*	*219*	*Ser (S)*
g.5276G>A (R)	**G**AG	Glu (E)	**A**AG	222	Lys (K)
g.5314G>A (R)	*AA**G***	*Lys (K)*	*AA**A***	*235*	*Lys (K)*
g.5330G>A (R)	**G**AC	Asp (D)	**A**AC	240	Asn (N)
**Exon 7**					
g.6961–6966 Ins TGCCAA	**-**	-	**TGCCAA**	298–299	Asn (N) Ala (A)

(*) Numbering of SNPs submitted in dbSNP/NCBI database; (**) The localization of substitution in the 387 aa pPAG2 precursor (numeration before removal of the conserved 15 aa signal peptide).

## Data Availability

The data presented in this study are available in this article.
